# Three-step surgical treatment of aortoesophageal fistula after thoracic endovascular aortic repair: A case report

**DOI:** 10.1016/j.ijscr.2019.10.066

**Published:** 2019-11-03

**Authors:** Atsushi Kamigaichi, Yoichi Hamai, Manabu Emi, Yuta Ibuki, Shinya Takahashi, Keijiro Katayama, Tomokuni Furukawa, Morihito Okada

**Affiliations:** a Department of Surgical Oncology, Hiroshima University, Hiroshima, Japan; b Department of Cardiovascular Surgery, Graduate School of Medicine, Hiroshima University, Hiroshima, Japan; c Cardiovascular Center, Department of Cardiovascular Surgery, Akane-Foundation Tsuchiya General Hospital, Hiroshima, Japan

**Keywords:** Aneurysm, Esophagus, Fistula, Stent-graft, Thoracic endovascular aortic repair

## Abstract

**Introduction:**

Aortoesophageal fistula (AEF) is a fatal complication results in sudden massive hematemesis. Although thoracic endovascular aortic repair (TEVAR) is an established method of treating aortic aneurysms or aortic dissection, the number of AEF after TEVAR is recently increasing due to the spread of TEVAR. However, the therapeutic strategy for AEF remains controversial.

**Presentation of case:**

We describe a 71-year-old man with Stanford B aortic dissection and aortic aneurysm rupture treated by TEVAR who developed AEF between the thoracic aorta and upper thoracic esophagus 20 months thereafter. We applied a three-step surgical procedure for this patient comprising resection of the esophagus as the infectious source, removal of an aortic aneurysm with stent-graft and replacement of the aorta, and final reconstruction of the esophagus. Thereafter, the patient resumed oral intake and has remained relapse-free for 24 months without adverse events.

**Discussion:**

Previous reports have described simultaneous resection of the esophagus and aortic stent-graft via a left thoracotomy followed by a two-step surgical reconstruction of the esophagus. We applied a three-step procedure consisting of resections of the esophagus and aortic stent-graft on separate occasions followed by esophageal reconstruction in this patient. The first procedure in the three-step approach is less stressful than that of the two-step approach.

**Conclusion:**

The three-step surgical approach to treating AEF after TEVAR resulted in a good outcome for this patient. Thus, this surgical strategy is a useful option for treating AEF after TEVAR.

## Highlights

• The number of aortoesophageal fistula (AEF) after thoracic endovascular aortic repair (TEVAR) is recently increasing due to the spread of TEVAR.
• AEF is a rare but fatal disease, and only surgery can save the life of patients with AEF after TEVAR.
• The therapeutic strategy for AEF after TEVAR remains controversial.
• The three-step surgical approach described herein could be a useful therapeutic option for AEF after TEVAR.

## 1 Introduction

Endovascular techniques were first reported for the abdominal aortic aneurysms by Parodi in 1991 [1]. The first successful outcome of treatment for thoracic aortic aneurysm reported by Dake in 1994, was referred to as thoracic endovascular aortic repair (TEVAR) [2]. Since then, TEVAR has become an established treatment for aortic aneurysms or aortic dissection because it is minimally invasive and therapeutic outcomes are good [3]. On the other hand, TEVAR is also associated with several complications, including paraplegia, renal failure, stroke, post-implantation syndrome, device migration and aortoesophageal fistula (AEF) formation [[4], [5], [6]].

The formation of an AEF after TEVAR was originally reported in 1998 by Norgren [7] and the number of reports describing AEF has increased as TEVAR applications have widened and post-treatment followup periods have lengthened. Aortoesophageal fistulae develop after TEVAR in 1.7%–1.9% of patients at a median of 11.6 months [8,9]. The main causes of death are fatal bleeding, mediastinitis and sepsis [9]. The reported mortality rates after surgical and conservative therapy for AEF after TEVAR are 64% and 100%, respectively [5]. Therefore, the prognosis of AEF after TEVAR is almost as poor as that of AEF arising in the absence of TEVAR, and only surgery can save the life of patients with AEF after TEVAR. However, treatment strategies including surgical approaches remain controversial. We describe a patient with AEF after TEVAR who was treated via a three-step surgical approach with a good outcome. This work has been reported in line with the SCARE criteria [10].

## 2 Case presentation

**Fig. 1:**
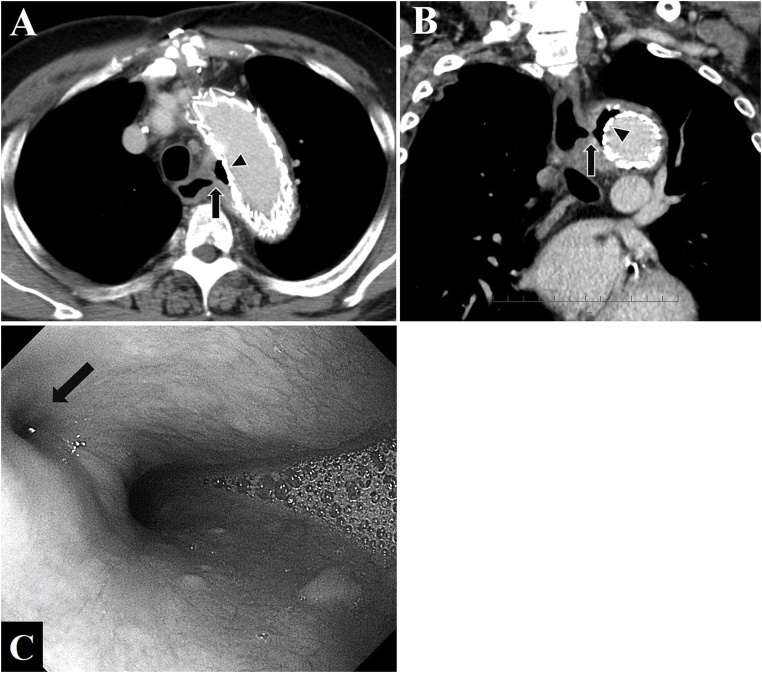
A, B. Computed tomography images of fistula between aorta and esophagus (arrow) and free air surrounding aortic stent-graft (arrowhead). A, axial image. B, coronal image. C. Upper gastrointestinal endoscopy shows ulcer on left lateral wall of upper thoracic esophagus (arrow).

**Fig. 2:**
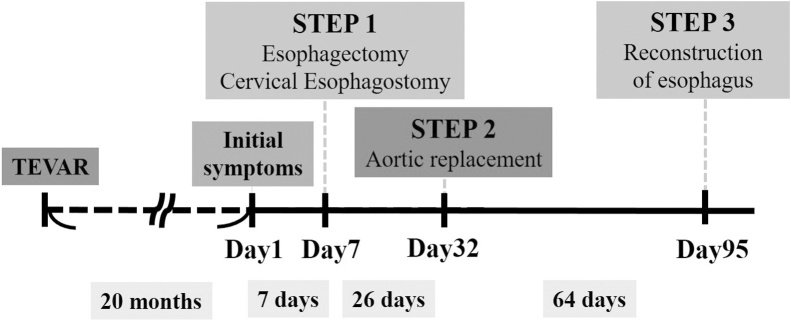
Clinical course of the patient with aortoesophageal fistula after thoracic endovascular aortic repair.

A 71-year-old man with a history of TEVAR for Stanford B aortic dissection and aortic aneurysm rupture 20 months ago presented at a local medical clinic with fever over 38 °C. Laboratory findings revealed elevated infectious indicators, and he was prescribed with antibiotics. However, he presented at his primary care hospital one week later without symptomatic improvement. Contrast-enhanced computed tomography at that time identified a fistula between the esophagus (Fig. 1A and B) and an aortic aneurysm, and upper gastrointestinal endoscopy revealed an esophageal ulcer (Fig. 1C). Therefore, he was diagnosed with AEF after TEVAR and immediately transferred to our hospital for surgical therapy. On admission, he did not have hematemesis and was hemodynamically stable. He required emergency surgery to the control the spread of infection and prevent fatal bleeding. We planned a three-step surgical approach (Fig. 2).

**Fig. 3:**
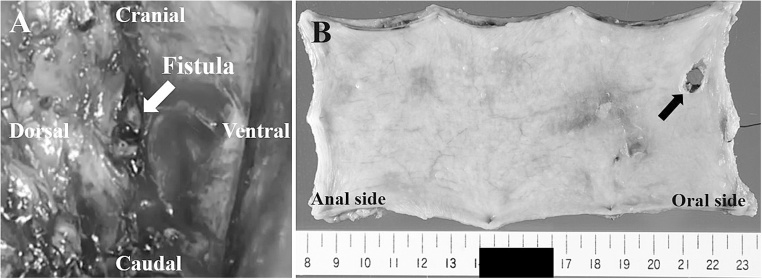
Intraoperative and gross findings of fistula. A: Intraoperative findings of fistula in aortic wall after esophageal resection (arrow). Blood oozed only from fistula. B: Gross findings of esophagus show fistula with maximum diameter of 1.0 cm (arrow).

The first step of the procedure on the day of admission comprised esophagectomy via a right thoracotomy at the fourth intercostal space with the patient in the left lateral position. We cut the esophagus above the AEF and diaphragm and resected part of it that also included the AEF. Intraoperative findings revealed extensive inflammation of the mediastinal tissue and leakage of infected old blood from the aortic fistula without massive bleeding (Fig. 3A). We placed drains in the right thoracic cavity and the mediastinum beside the aortic fistula. The patient was then placed in the supine position and the residual esophagus was brought to the left cervical region as an esophagostomy. A feeding jejunostomy tube was then placed via a small abdominal incision. He was admitted to the surgical intensive care unit thereafter, and infection control was started by abscess draining and antibiotic administration. Gross findings of the resected esophagus showed a perforation site with a maximum diameter of 1.0 cm (Fig. 3B).

No bacteria were identified in blood culture; however, *Klebsiella pneumonia* and *Prevotella melaninogenic* were identified in mediastinal tissue culture. Antibiotics, abscess drainage and pleural lavage were performed, however they did not completely improve the inflammatory response after the first surgery. Therefore, we removed residual infected foci as soon as the patient’s status became stabilized. The second step of the procedure was implemented one month later to remove the thoracic aortic aneurysm and artificial stent-graft and to restore the aorta in situ with a synthetic vascular prosthesis through a left thoracotomy. The prosthesis was infiltrated with rifampicin before graft replacement to prevent repeated infection. Thereafter, the inflammatory response and the general status of the patient gradually improved under antibiotics, drainage and pleural lavage. He developed strength through postoperative rehabilitation and enteral nutrition management.

Three months after the second step, the third step addressed the esophageal defect. A narrow gastric tube fashioned by laparotomy was brought up through the ante-thoracic route, and cervical esophagogastrostomy proceeded. The patient recovered uneventfully, resumed oral intake and was discharged on postoperative day 37. The patient remains free of disease and adverse events at 24 months after completing the three-step procedure.

## 3 Discussion

Although AEF is rare, it is fatal with sudden massive hematemesis. However, the number of reported cases which can get back into society has been gradually increasing since a life-saving case by the surgical procedure was reported for the first time in 1983 [11]. The causes of AEF are aortic aneurysm (50%), esophageal cancer (17%), esophageal foreign body (20%) and others including trauma and surgical complications (13%) [12].

Recent reports have indicated that TEVAR can cause AEF [6,8,13]. Several proposed mechanisms of AEF development after TEVAR include infection of a stent-graft and aortic aneurysm, direct erosion of a stent-graft through the aorta into the esophagus, necrosis due to continuous pressure from an aortic stent-graft and large aneurysm and ischemic esophageal necrosis due to occlusion of the esophageal artery that feeds the esophagus [6,8,13] More AEF after TEVAR is predictable due to the recent broadening of TEVAR applications.

Symptoms of AEF comprise not only hemorrhage or severe chest/back pain, but also vague non-specific symptoms such as fever and an elevated inflammatory response [[14], [15], [16]]. The frequency of massive bleeding associated with AEF after TEVAR is relatively low because the fistula is located between the esophagus and false lumen of aorta after TEVAR. This can delay initial treatment as in our patient. When patients with a history of TEVAR present with non-specific symptoms, AEF should be considered.

The control of infection and fatal bleeding is mandatory to save the lives of patients with AEF after TEVAR and sources of infection such as the esophagus, aortic wall and artificial stent-graft must be removed. Thereafter, antibiotics and sustainable drainage with lavage is required for continued infection control. Moreover, re-implantation of a synthetic vascular prosthesis with protection against re-infection, such as a synthetic graft infiltrated with antibiotics and omental packing, are necessary for revascularization [17].

Previous reports have described simultaneous resection of the esophagus and aortic stent-graft via a left thoracotomy followed by a two-step surgical reconstruction of the esophagus [17,18]. Here, we applied a three-step procedure consisting of resections of the esophagus and aortic stent-graft on separate occasions followed by esophageal reconstruction, because massive bleeding did not occur in AEF after TEVAR in this patient. The first procedure in the three-step approach is less stressful than that of the two-step approach. Furthermore, we could restore the aorta during the second procedure using a synthetic vascular prosthesis under conditions of considerable infection control. Thereafter, esophageal reconstruction can be planned as the third step after total infection control and adequate improvement in the general physical status of a patient with AEF. The general status of such patients is often too poor to endure highly invasive surgery. Therefore, we considered the need to improve safety as much as possible during each highly invasive step.

The shortcoming of the three-step surgical approach is the possibility of difficult infection control after the first step due to an unresected infected aorta and risk of bleeding from the fistula. Therefore, the second step might need to be implemented as soon as possible if difficulties are encountered with infection control or bleeding. The main advantage of two-step surgery is better infection control because of complete removal of the infected tissue. From this point of view, two-step surgical approach may be more suitable for patients who can endure high operative stress.

Reports describing AEF after TEVAR remain scant and optimal therapeutic strategies remain controversial. Here, we found that a three-step surgical approach improved the safety of each step of the procedure by reducing surgical stress. This resulted in a good outcome for this patient with AEF. Thus, this surgical strategy might be a useful option for treating AEF after TEVAR.

## 4 Conclusions

Optimal therapy could save the lives of patients with AEF after TEVAR. Treatment strategies remain controversial, but we feel that the three-step surgical approach described herein could be a useful therapeutic option for AEF after TEVAR.

## Declaration of Competing Interest

All authors have no conflict of interest.

## Sources of funding

No funding was received.

## Ethical approval

The Institutional Review Board at Hiroshima University – This investigation is exempt from ethical approval at our institution.

## Consent

The patient provided written, informed consent to the publication of this case report.

## Author contribution

AK wrote the manuscript. YH, YI and ME supervised writing the manuscript. All authors were part of the surgical team that treated this patient. All authors read and approved submission of the final manuscript.

## Registration of research studies

Not applicable.

## Guarantor

Atsushi Kamigaichi.

Morihito Okada.

## Provenance and peer review

Not commissioned, externally peer-reviewed
